# Association of Aortic Diameters with Coronary Artery Disease Severity and Albumin Excretion

**DOI:** 10.1155/2015/857628

**Published:** 2015-08-27

**Authors:** Bülent Özdemir, Ali Emül, Levent Özdemir, Saim Sağ, Murat Biçer, Ali Aydınlar

**Affiliations:** ^1^Department of Cardiology, Uludağ University, Bursa, Turkey; ^2^Şevket Yılmaz Government Research and Training Hospital, Bursa, Turkey; ^3^Department of Public Health, Cumhuriyet University, Sivas, Turkey; ^4^Tıp Fakültesi Kalp, Damar Cerrahisi Anabilim Dalı, Uludağ Üniversitesi, Görükle Kampüsü, Nilüfer, 16059 Bursa, Turkey

## Abstract

*Introduction*. Aortic diameters, aortic distensibility, microalbuminuria, coronary artery disease which are all together related to vascular aging are investigated in this paper. *Methods*. Eighty consecutive nondiabetic patients undergoing elective coronary angiography were enrolled into the study. Systolic and diastolic aortic diameters, aortic distensibility, CAD severity by angiogram with the use of Gensini scoring, and albumin excretion rates were determined. *Results*. Cases with CAD had significantly larger systolic (30,72 ± 3,21 mm versus 34,19 ± 4,03 mm for cases without and with CAD, resp.) and diastolic aortic diameters measured 3 cm above aortic valve compared to patients without CAD (33,56 ± 4,07 mm versus 29,75 ± 3,12 mm). The systolic and diastolic diameters were significantly higher in albuminuria positive patients compared to albuminuria negative patients (*p* = 0.017 and 0.008, resp., for systolic and diastolic diameters). *Conclusion*. In conclusion aortic diameters are increased in patients with coronary artery disease and in patients with microalbuminuria. In CAD patients, systolic blood pressure, pulse pressure, aortic systolic and diastolic pressure, and albumin excretion rate were higher and aortic distensibility was lower.

## 1. Introduction

Aortic diameters are related to coronary artery disease. In a recent study, ascending aorta, aortic arch, distal thoracic aorta, and abdominal aorta diameters measured during autopsies of subjects that died of cardiovascular disease were significantly bigger than those of the subjects who died from other causes [1]. Aortic distensibility is a characteristic of the vessel wall and is related somewhat to contraction and dilation of the aorta. The definition can be equated as maximum change in area/(minimum area × pulse pressure). It can be measured anywhere through the course of the aorta. Many studies reported these changes during the cardiac cycle in both the ascending aorta and the descending aorta [2].

Coronary artery disease severity and extent determined by modified Gensini score were predicted by distal descending atheroma burden, ascending aorta distensibility, carotid flow mediated dilatation, and atheroma class in a magnetic resonance imaging study [3].

Association of aortic stiffness and microalbuminuria has been the focus of many studies [4–7]. In patients with hypertension, microalbuminuria and mildly reduced creatinine clearance were independently related to aortic stiffness which was calculated with carotid and femoral pulse [8]. Aortic distensibility is related to coronary flow reserve and coronary flow restoration with percutaneous coronary interventions improves aortic distensibility and coronary flow velocity reserve [9, 10].

The rate of having coronary heart disease among renal failure patients is quite high [11]. The same risk factors may affect both renal and cardiac functions and vasculature. End stage renal disease patients' coronary artery disease risk is 10 times higher compared to high-risk patients of Framingham's study [12]. Microalbuminuria was used as a marker for diabetic nephropathy and lately for cardiovascular morbidity and mortality [13, 14]. Also the association of the severity of coronary artery disease with microalbuminuria presence was reported in another study by Khan et al. [15]. In a study that utilised coronary computed tomography angiography, association of presence, extent, and severity of coronary artery disease in asymptomatic patients with type 2 DM with microalbuminuria was investigated. In that study patients with microalbuminuria had more severe coronary lesions. Microalbuminuria clearly predicted high coronary artery disease risk and worse clinical outcomes [16].

## 2. Materials and Methods

Eighty consecutive patients undergoing elective coronary angiography in the Cardiology Department of the Applied Research Centre for Health of Uludağ University were enrolled into the study. The angiograms were performed with use of Siemens cardiac catheterization unit in the Hemodynamics Laboratory of the Cardiology Department. This was a prospective study in which the aortic diameters and aortic elasticity were determined along with the Gensini score that allowed assessing the severity of CAD [17]. A Gensini score greater than 20 was defined as a high Gensini score.

**Figure 1:**
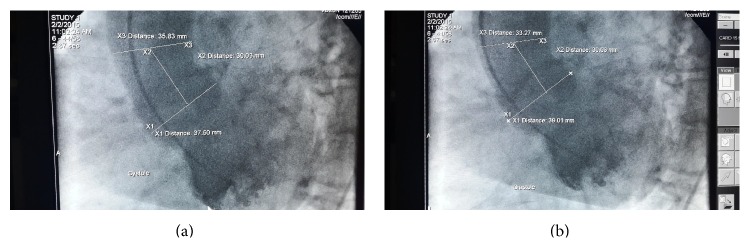
Aortic diameters are measured 3 cm above the aortic orifice. The illustrations cover systole (a) and diastole (b).

Systolic and diastolic diameters were measured 3 cm above the aortic valve (in cm) (Figures 1(a) and 1(b)). Aortic distensibility was calculated as follows: 2 × (change in aortic diameter)/(diastolic aortic diameter) × (change in aortic pressure), where change in aortic diameter equals systolic minus diastolic aortic diameter, and change in aortic pressure equals systolic minus diastolic aortic pressure [18, 19]. Aortic pulse pressure was calculated as systolic aortic pressure minus diastolic aortic pressure. Hypertension was defined as having blood pressure greater than or equal to 140/90 mmHg or being on treatment.

The coronary angiograms were evaluated. Chi-Square Test was used for classified variables and Mann-Whitney* U* test was used for comparison of two groups. The statistical analyses were performed by use of SPSS data Manager Software system. Statistical significance was assumed in case of a *p* value <0.05. The results are expressed as mean ± standard deviation.

The patients were evaluated by dividing into two groups according to having CAD and also having albuminuria. The comparisons were made by categorising the cases as having no CAD, mild CAD, and severe coronary artery diseases. Also comparisons were made according to having no, moderate, and severe albumin excretion rates.

## 3. Results and Discussion

The characteristics of the patients are given in Table 1. When the cases were divided into two according to having CAD or not having CAD, aortic dimensions both in systole and diastole were significantly different. When systolic aortic diameter was compared, cases with CAD had significantly larger systolic aortic diameter (30,72 ± 3,21 mm versus 34,19 ± 4,03 mm for cases without and with CAD, resp.). Also the same was true for the aortic diastolic diameters measured 3 cm above aortic valve with significantly bigger aortic diameter for the CAD patients compared to patients without CAD (33,56 ± 4,07 mm versus 29,75 ± 3,12 mm). The aortic distensibility showing the elastic properties of the aorta was significantly smaller in CAD patients (*p* = 0.013) (Table 2). Also in CAD patients the systolic blood pressure was significantly higher compared to cases without CAD (127,4 ± 14,7 mmHg versus 117,1 ± 11,9 mmHg; *p* = 0.03). However the diastolic blood pressure failed to show a significant difference. Pulse pressure and proteinuria level also were significantly higher in CAD patients (Table 2).

In Table 3 the comparison of the albuminuria positive and albuminuria negative groups is given. Systolic pressure and diastolic pressure were indifferent in albuminuria positive and albuminuria negative patients. Pulse pressure was significantly higher in albuminuria positive patients. Albuminuria presence did not cause significant difference in aortic distensibility among groups. But both the systolic and diastolic diameters were significantly higher in albuminuria positive patients compared to albuminuria negative patients (*p* = 0.017 and 0.008, resp., for systolic and diastolic diameters).

Grouping the cases by <50 years of age and ≥50 years of age showed that rate of presence of hypertension was significantly higher in the cases belonging to the latter group (25% versus 66.2%, resp., with a *p* value <0.05). However, rate of presence of albuminuria was not significantly different between the groups of cases with <50 and ≥50 years of age. Also grouping of cases according to a cut-off of age of 50 years showed that rate of having CAD in the cases with age of 50 years or older was significantly higher compared to others. Also only 3,1% of cases with significant coronary artery disease according to Gensini scoring were younger than 50 years of age. A cut-off value of 50 mmHg for pulse pressure significantly showed that a higher level of albumin excretion was the case for cases which had a pulse pressure of 50 mmHg or higher compared to others (*p* < 0.05).

**Figure 2:**
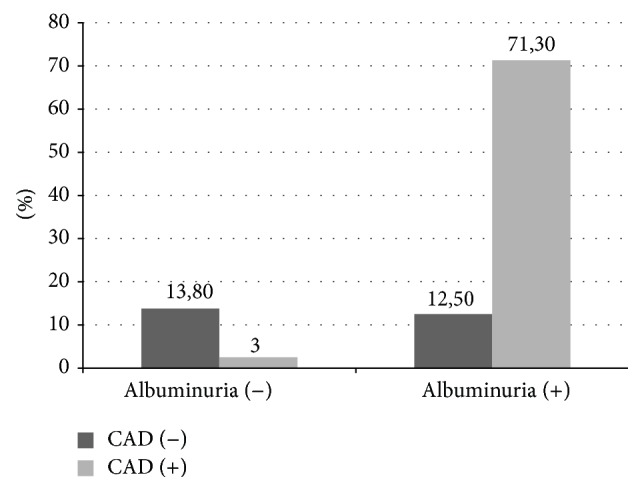
Percent distribution of all the cases (*n* = 80) according to presence of CAD and albuminuria. Albuminuria presence was significantly associated with presence of coronary artery disease (*p* < 0,001).

When cases with CAD were compared to cases without CAD the mean albumin excretion rate was significantly lower (*p* < 0.05). However, albumin excretion rate did not differ between cases with mild CAD and cases with severe CAD patients (*p* > 0.05). When patients were grouped into having microalbuminuria or having CAD and compared accordingly, presence of albuminuria predicted presence of CAD (Figure 2).

Presence of CAD is significantly related to aortic diameters [1]. In the study by Milan et al. all the studied segments of aorta were significantly larger in the group with cardiovascular disease. A tendency to decrease in the size of aorta from ascending to abdominal aorta segments was also noted in the study that involved autopsy subjects. These results were consistent with the results in this paper. In cases with hypertension dilatation of the ascending aorta is more common [20]. In a study by Giannattasio et al., arterial distensibility in patients with type I (insulin-dependent) diabetes mellitus without macrovascular complication was evaluated. In their study arterial distensibility was lower in a consistent manner compared to the control group. Noting increased radial and carotid artery thicknesses, they stated that the changes were more pronounced in patients with microalbuminuria, retinopathy, or neuropathy. Interestingly, the arterial wall stiffening and thickening was seen in the absence of diabetic complications, suggesting those being an early marker of vascular damage [21]. Arterial stiffness also is associated with premature coronary artery disease occurrence [22]. Being consistent with the result of our study there are reports saying that in CAD patients the aortic distensibility is markedly reduced. Ascending aorta stiffness index is associated with coronary artery disease in hypertensive patients [23].

In our study albuminuria did not affect aortic distensibility but aortic diameters were significantly higher in the albuminuria positive patients. However, in a study by Cuspidi et al. microalbuminuria was not associated with abdominal aortic diameter [24]. Also endothelial dysfunction is associated with microalbuminuria as reported 2 decades ago by Pedrinelli et al. In their study von Willebrand Factor antigen concentrations that showed presence of endothelial dysfunction were higher in hypertensive patients with microalbuminuria compared to other groups [25]. Coronary artery disease is considered as an important complication of type 2 diabetes mellitus. Guo et al. evaluated the correlation of urinary albumin excretion rate with the coronary heart disease severity and incidence, in patients with type 2 diabetes mellitus, aged 60 years or older. They reported that urinary albumin excretion rate was independently correlated with coronary artery disease in patients with type 2 diabetes mellitus. Also the severity of coronary artery disease determined with Gensini scoring was independently associated with urinary albumin excretion rate [26]. However in our study the microalbuminuria did not differ between mild and severe coronary artery disease patients according to Gensini scoring. That makes us think that microalbuminuria helps us to detect presence of CAD but fails to show the extent of the disease. But we have to admit that these subjects need further investigation.

## 4. Conclusion

In conclusion aortic diameters are increased in patients with coronary artery disease and in patients with microalbuminuria. In CAD patients, systolic blood pressure, pulse pressure, aortic systolic and diastolic pressure, and albumin excretion rate were higher and aortic distensibility was lower.

## Figures and Tables

**Table 1:** Characteristics of the patients.

	Male	%	Female	%	Total
Gender	49	61,3	31	38,7	80

	Present	%	Absent	%	

Obesity	21	26,3	59	73,7	80
Smoking	38	47,5	42	52,5	80
Hypertension	48	60,0	32	40,0	80
Dyslipidemia	37	46,3	43	53,8	80

Age (years)	59,9 ± 10,0				
Height (cm)	165,8 ± 6,9				
Weight (kg)	75,9 ± 9,5				
BMI (kg/m^2^)	27,6 ± 3,9				

Serum levels of biochemical markers					

Total cholesterol (mg/dL)	183,4 ± 47,9				
LDL cholesterol (mg/dL)	113,5 ± 38,3				
HDL cholesterol (mg/dL)	44,0 ± 13,6				
Triglyceride (mg/dL)	140,7 ± 68,6				
Urea (mg/dL)	36,9 ± 12,8				
Creatinine (mg/dL)	0,98 ± 0,23				
Uric acid (mg/dL)	5,58 ± 1,82				
Haemoglobin (g/dL)	12,84 ± 1,96				

Aortic measurements					

Systolic blood pressure (mmHg)	124,7 ± 14,6				
Diastolic blood pressure (mmHg)	74,9 ± 8,9				
Pulse pressure (mmHg)	49,8 ± 10,8				
Mean aortic pressure (mmHg)	100,1 ± 14,0				
Aortic distensibility	2,88 ± 2,21				
Aortic systolic diameter (mm)	33,2 ± 4,11				
Aortic diastolic diameter (mm)	32,5 ± 4,1				

**Table 2:** Aortic measurement and albuminuria levels according to CAD presence.

	Coronary artery disease negative (*n* = 21)			Coronary artery disease positive (*n* = 59)			*p* value
	Median	(min–max)	IQR	Median	(min–max)	IQR	
^*^Systolic blood pressure	117	95–135	19	127	97–184	19	0,003
^*^Diastolic blood pressure	75	64–94	11,50	74	57–98	12	0,45
^*^Pulse pressure	43	26–65	13	52	32–78	13	0,003
^*^Mean aortic pressure	100	70–120	12	100	73–133	17	0,12
Aortic distensibility	3,02	0,99–14,40	3,80	2,27	0,32–11,20	1,60	0,013
Aortic systolic diameter (mm)	30,61	25,81–38,10	5,41	33,52	27,32–48,16	6,30	<0,001
Aortic diastolic diameter (mm)	29,47	25,46–36,83	5,62	33,02	26,73–47,06	6,46	<0,001
Proteinuria level	12	4–256	125	170	5–1931	161	<0,001

^*^mmHg; IQR: interquartile range.

**Table 3:** Comparison of the groups according to presence of albuminuria.

	Proteinuria negative (*n* = 13)			Proteinuria positive (*n* = 67)			*p* value
	Median	(min–max)	IQR	Median	(min–max)	IQR	
^*^Systolic blood pressure	117	103–136	20	126	95–184	16	0,11
^*^Diastolic blood pressure	77	64–94	10	74	57–98	10	0,13
^*^Pulse pressure	41	26–51	9	52	32–78	13	0,01
^*^Mean aortic pressure	100	73–120	13,5	100	70–133	17	0,24
Aortic distensibility	2,93	1,56–14,40	3,70	2,44	0,32–11,20	1,98	0,14
Aortic systolic diameter (mm)	30,61	26,55–38,31	5,60	33,11	25,81–48,16	6,30	0,01
Aortic diastolic diameter (mm)	29,18	26,10–37,71	5,84	32,78	25,46–47,06	6,62	0,008

^*^mmHg; IQR: interquartile range.
